# Tumor control probability reduction in gated radiotherapy of non‐small cell lung cancers: a feasibility study

**DOI:** 10.1120/jacmp.v16i1.4444

**Published:** 2014-10-16

**Authors:** R. Alfredo Siochi, Yusung Kim, Sudershan Bhatia

**Affiliations:** ^1^ Department of Radiation Oncology University of Iowa Hospitals and Clinics Iowa City IA USA

**Keywords:** tumor control probability, non‐small cell lung cancer, gated radiotherapy

## Abstract

We studied the feasibility of evaluating tumor control probability (TCP) reductions for tumor motion beyond planned gated radiotherapy margins. Tumor motion was determined from cone‐beam CT projections acquired for patient setup, intrafraction respiratory traces, and 4D CTs for five non‐small cell lung cancer (NSCLC) patients treated with gated radiotherapy. Tumors were subdivided into 1 mm sections whose positions and doses were determined for each beam‐on time point. (The dose calculation model was verified with motion phantom measurements.) The calculated dose distributions were used to generate the treatment TCPs for each patient. The plan TCPs were calculated from the treatment planning dose distributions. The treatment TCPs were compared to the plan TCPs for various models and parameters. Calculated doses matched phantom measurements within 0.3% for up to 3 cm of motion. TCP reductions for excess motion greater than 5 mm ranged from 1.7% to 11.9%, depending on model parameters, and were as high as 48.6% for model parameters that simulated an individual patient. Repeating the worst case motion for all fractions increased TCP reductions by a factor of 2 to 3, while hypofractionation decreased these reductions by as much as a factor of 3. Treatment motion exceeding gating margins by more than 5 mm can lead to considerable TCP reductions. Appropriate margins for excess motion are recommended, unless applying daily tumor motion verification and adjusting the gating window.

PACS numbers: 87.55.dk, 87.57.Q‐

PACS numbers: 87.55.dk, 87.57.Q-

## I. INTRODUCTION

Respiratory motion can cause lung tumor underdoses and healthy tissue overdoses when not accounted for in radiotherapy (RT) planning. One technique for managing tumor motion is gated RT. In gated RT, the beam is turned on once the tumor is completely inside the opening that defines the radiation field. As soon as any portion of the tumor goes outside the field, radiation is turned off.

Fields are designed to contain minimal (< 1 cm) tumor motion. However, monitors used as signals to control irradiation may not measure tumor motion, but rather some surrogate, such as marker block motion or strain gauge (SG) pressure. Surrogate signals, tumor motion, and their relationships may vary between fractions. Thus, tumors could move beyond the motion margins included in the treatment fields.[1] Resulting cold spots can reduce the tumor control probability TCP.[2]

[a] Corresponding author: R. Alfredo Siochi, Department of Radiation Oncology, University of Iowa Hospitals and Clinics, 749 Arlington Dr, Iowa City, IA 52245, USA; phone: (319) 541 0016; fax: (319) 356 1530; email: alf@siochi.info

To evaluate the effect of excess motion on TCP, we calibrated the surrogate against tumor motion via pretreatment cone-beam CT (CBCT) projections[3] and SG signals. While intrafractional 4D CBCT scans could verify tumor motion,[4] they do not provide tumor positions when the treatment beam is on. We overcome the lack of tumor position information by using SG readings recorded at beam-on times and converting them to tumor positions. We then determine delivered doses to various locations in the tumor for the entire treatment course and calculate the TCP. For a group of patients with varying degrees of motion, one expects to see variations in their TCPs. The analysis of such variations can provide qualitative guidelines for identifying patients whose excess motion could compromise their treatment. While such an analysis has been performed with treatment planning simulations of non-gated RT patients,[5] the methodology presented here uses the recorded tumor motion at treatment time for gated RT patients.

All studies to date have only used planned dose distributions to calculate the TCP. The use of delivered doses will allow us to improve the current TCP models. While it would be ideal to have an implanted *in vivo* dosimeter to get actual delivered doses, it is impractical. In this study, we evaluate the feasibility of estimating the delivered doses using CBCT and strain gauge data recorded during treatment delivery, calculate the TCPs, and compare the results against TCPs estimated with current models for local progression-free survival.

## II. MATERIALS AND METHODS

The experiments consist of several parts. We used patient strain gauge recordings (as a respiratory monitor), treatment planning four-dimensional CT (4D CT) scans, and daily cone-beam CT (CBCT) images to estimate tumor position as a function of strain gauge values. The strain gauge recordings also included information from the linear accelerator about when the radiation was turned on and off. The recorded beam status allowed us to estimate where the tumor was when the radiation was turned on. To estimate the dose distribution delivered to portions of the tumor, we used a simple model of the radiation beam: a central uniform dose area with a penumbra at each edge of the beam. We tested the simple model using a phantom with a Farmer chamber that moved according to a well-characterized sinusoidal pattern. The test method allowed us to compare the dose calculation for the phantom against the measured dose and validate the utility of the simple beam model for making dose estimates. We then applied the beam model to calculate the patient's tumor dose distributions using the estimated tumor positions recorded during the treatment. Finally, we used the estimates of the dose distributions delivered to the tumor to determine the tumor control probability (TCP).

### A. 4D CT imaging

Four-dimensional computed tomography (4D CT) scans were acquired for five NSCLC patients (Table 1). 3D CT image sets for 10 respiratory phases were reconstructed from the 4D CT raw data by retrospectively analyzing the respiratory trace acquired during the 4D CT scan. The respiratory trace was acquired using the AZ733V strain gauge system (Anzai Medical Co., Tokyo, Japan) connected to the Siemens Biograph PET/CT scanner (Siemens Medical Solutions, Inc, Malvern, PA). The PET/CT scanner used the respiratory information to sort images from different phases. The 10 phases that were reconstructed were full exhale, full inhale, four mid-exhale scans, and four mid-inhale scans. The mid-inhale and mid-exhale scans were reconstructed from data acquired when the respiratory traces were at 20%, 40%, 60%, and 80% of the way from full exhale to full inhale (for the mid-inhale scans) and from full inhale to full exhale (for the mid-exhale scans).

We analyzed the ten 3D CT images by playing them in a movie loop to look for the phases that corresponded to 1 cm of motion. Because of the nonlinear relationship between strain gauge values and tumor positions, the phase corresponding to the specified tumor displacement during inhale can be different from the one during exhale. For example, in one patient's case we used

Table 1. Treatment information.

| *Patient* | *1* | *2* | *3* | *4* | *5* |
|---|---|---|---|---|---|
| Fraction size (Gy) | 16 | 16 | 6 | 5 | 1.8 |
| Number of fractions | 3 | 3 | 10 | 10 | 37 |
| Tumor, SI (mm) | 30 | 31 | 74 | 15 | 50 |
| Target for ITV | GTV | GTV | GTV | GTV | CTV |
| Motion margin (mm) | 4 | 30 | 6 | 10 | 10 |
| PTV margin (mm) | 3 | 5 | 10 | 10 | 0 |
| Block margin (mm) | 5 | 5 | 5 | 5 | 5 |
| Craniocaudal field size (mm) | 50 | 85 | 110 | 55 | 70 |
| Mean excess motion (mm) | 2.9 | < 0 | 10.1 | <0 | 8.8 |

the 3D CT data corresponding to 40% mid-exhale and 20% mid-inhale to include about 1 cm of motion in the treatment plan. The phase information was recorded in the patient's prescription, since we set the strain gauge gating window to the prescription phases for treatment.

While the difference in mid-exhale and mid-inhale values seems large (20%), the difference is a consequence of reconstructing only 10 phases. If we had reconstructed more, the difference would have been less. Also, the amount of motion on the mid-exhale and mid-inhale scans is not the same. The only requirement for selecting phases is that the motion be as large as possible without exceeding 1 cm. In the previously cited patient example, the 40% mid-exhale motion was about 9 mm, while the 20% mid-inhale motion was about 6 mm.

## B. Treatment plans

Table 1 shows the treatment information for five NSCLC patients who received gated radiotherapy (two stereotactic body RT (SBRT), two hypofractionated, and one standard fractionation). The data were gathered retrospectively in accordance with our Institutional Review Board Project #200707726.

To create contours for the treatment plan, we selected phases from the 4D CT image dataset that represented the greatest extent of motion. The selected phases always included the full exhale CT and the full inhale CT, as well as mid-inhale and mid-exhale phases where the tumor is displaced 1 cm from its position at full exhale. Sometimes we selected additional mid-inhale or mid-exhale phases if they had more motion in the antero–posterior or lateral directions. We then contoured the gross tumor volume (GTV) in the selected phases. Taking the union of the GTVs produces the maximum target volume (MTV). Internal target volumes (ITVs) were defined by taking the union of either the GTVs or clinical target volumes (CTVs) (see Table 1) for the respiratory phases during which the patient receives radiation. The treatment phases include full exhale, mid-inhale, and mid-exhale phases where the tumor is displaced 1 cm or less from full exhale. Planning target volumes (PTVs) were created by expanding ITVs by the amount listed in Table 1 as the PTV margin, and a block margin was added to the PTV to define the gated treatment fields. Table 1 shows the various margins used in the study.

## C. Patient setup and treatment delivery

Megavoltage (MV) CBCT scans (Siemens MVision; Siemens Medical Systems, Erlangen, Germany) were acquired immediately prior to treatment with the patient positioned so that their setup marks aligned with the in-room isocenter lasers. The tumor in the (MV) CBCT scans was registered with the treatment planning contours of the maximum target volume. (An MV CBCT scan takes about 1 min, so it captures all the breathing motion. The MTV is the planning structure that contains all the breathing motion. It is the most similar to what is present in the MV CBCT.) During the registration process, a shift is sometimes introduced to align the volumes of interest. The shift represents the difference between the machine treatment isocenter and the desired location of the patient's treatment isocenter. The patient couch is shifted to align the patient and machine isocenters.

To set up the gating window for the patient, we followed our clinical practice of performing the initial adjustment to the strain gauge. The offset and gain of the strain gauge system were adjusted so that at full exhale the strain gauge reading was zero, while at full inhale the strain gauge reading was 100 (Fig. 1, right axis). Hence, all phase information represents (approximately) the percentage tumor displacement. For example, in one patient's case, a gating window from 40% exhale to 20% inhale for a total tumor displacement of 3 cm (from full exhale to full inhale) represented an ITV that includes displacements of up to 1 cm from FE, and an MTV that includes 3 cm of motion.

After the initial adjustment of the strain gauge, we set the gating windows on the strain gauge software application to match the strain gauge phases recorded in the patient's prescription. The strain gauge was also connected to the linear accelerator so that it could turn the beam on and off according to the prescription. In all cases, the beam was turned on at the prescribed mid-exhale phase, left on through full exhale, and turned off at the prescribed mid-inhale phase.

We operated the strain gauge system in two different modes. During treatment, we used the gating mode. In gating mode, we record strain gauge values and linear accelerator beam status, as well as control the linear accelerator beam. During CBCT acquisition, we used only the recording mode. The recording mode provided us the strain gauge values as a function of time. It also provides the linear accelerator beam-on and beam-off status as a function of time. For CBCT acquisition, the radiation beam-on and beam-off information corresponds to the

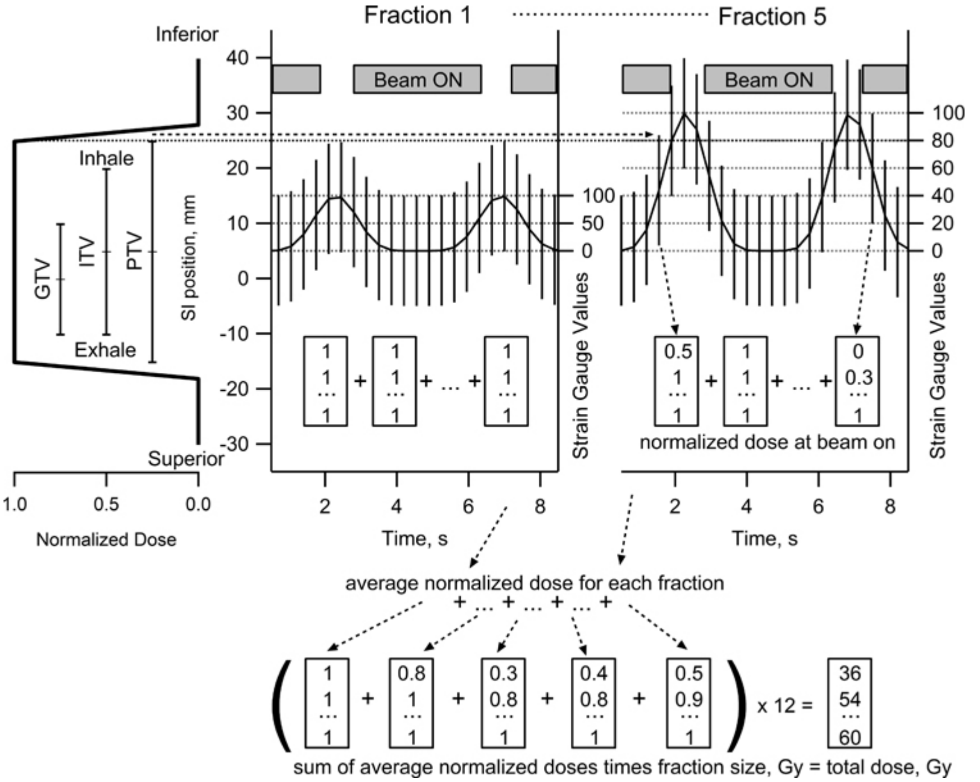

FIG. 1. Planned normalized dose distribution (top left) as a function of craniocaudal position; tumor position (top middle) as a function of time with a 5 mm inferior setup error for fraction 1; (top right) same as the middle except an excess motion of 15 mm has been added for fraction 5. Vertical lines on traces represent the tumor's craniocaudal extent, while rectangles with numbers represent arrays of normalized tumor dose distributions along the vertical lines. Right-side axes indicate strain gauge values. The numbers in rectangles are summed and divided by the number of beam on time points to get average normalized doses for each fraction. The sum (bottom) of the average normalized doses over all fractions is multiplied by the fraction size to get the total dose distribution in the tumor.

beam pulses that were used to acquire the sequence of 200 projections as the gantry rotated. Because we did not gate the CBCT acquisition, the CBCT images contained all the motion.

## D. Strain gauge calibration

Because a patient's breathing patterns can change from one day to the next and there can be variations in the way the strain gauge is set up, the same strain gauge value can represent different amounts of tumor motion on different treatment fractions. That means we need to calibrate the strain gauge on a daily basis. The calibration for a given fraction will enable us to calculate the tumor position that corresponds to a given strain gauge value for the given fraction. The calibration method we present here is simplified and manual, but it is sufficient for a feasibility study. It is also possible that for a long treatment fraction, the calibration may drift, and a recalibration in the middle of the fraction may be needed. See the Discussion section below for future enhancements to the method.

To calibrate the strain gauge, we determined the relationship of strain gauge values to tumor displacement from full exhale. The strain gauge file contained beam-on time stamps to associate a strain gauge value with the corresponding beam pulse that was used to acquire a projection in the CBCT raw data (Fig. 2, vertical bars, bottom graph). Identifying the tumor in the given projection provides the tumor position corresponding to the strain gauge value. However, since the tumor was difficult to visualize in several CBCT projections (Fig. 2, top row), we identified the ipsilateral hemidiaphragm apex instead (Fig. 2, middle graph).

We then converted the diaphragm apex positions to tumor positions by using the diaphragm-to-tumor motion ratio that was determined from the full exhale and full inhale phases of the planning 4D CT, where the tumor centroid and diaphragm apex displacements were readily measured. We also confirmed(3) that, for these patients, tumor motion in near AP projections of the CBCT raw data scaled with diaphragm motion by a constant ratio. (In the near AP projections of the CBCT, the tumor was much easier to visualize.) The diaphragm-to-tumor motion ratio for these patients ranged from 1 to 1.2. The diaphragm was a good surrogate for the tumor, consistent with the observations of Cervino et al.(6)

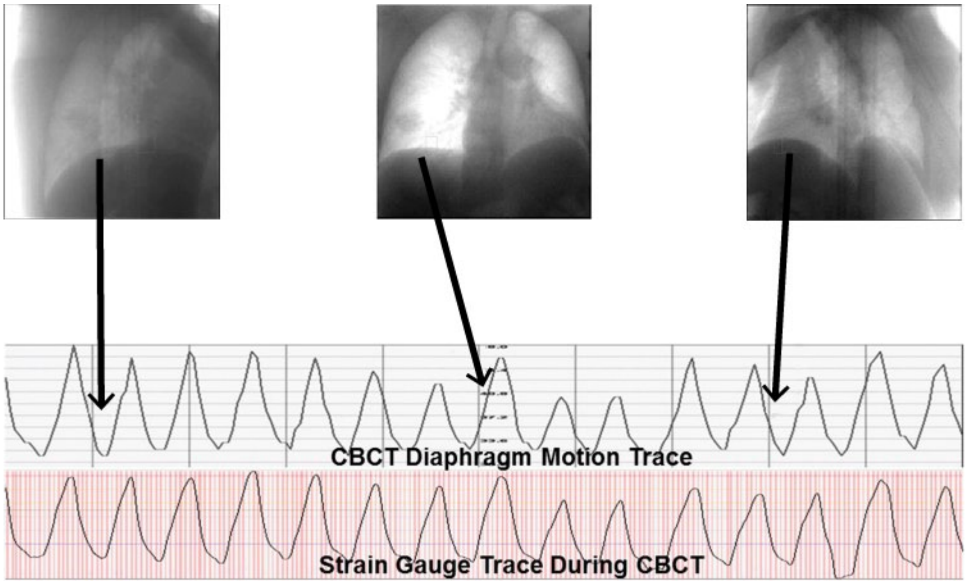

Fig. 2. Calibration of strain gauge values to diaphragm motion. The ipsilateral hemidiaphragm apex is identified in each projection of the CBCT raw data (top). Its craniocaudal position is identified to produce a CBCT diaphragm motion trace (middle). The diaphragm motion trace is compared to the strain gauge trace acquired during the CBCT acquisition (bottom). The light red vertical bars in the bottom row indicate when the projection images were acquired.

For our feasibility study, we used a linear relationship to determine the treatment time tumor displacement from the strain gauge value, S:

$$T = \frac{A}{R}S \tag{1}$$

$A$ is the average diaphragm apex displacement between full exhale and full inhale, divided by the average difference between strain gauge values at full exhale and full inhale, determined from the CBCT. $R$ is the diaphragm to tumor motion ratio. Using Eq. (1), we can convert all strain gauge values that were recorded for the treatment into tumor positions. We then use the tumor position as a function of time and compare that to the linear accelerator beam-on status as a function of time to determine where the tumor was when the radiation beam was turned on. We can use the tumor position information to estimate the dose distribution to the tumor.

## E. Dose calculation

To estimate the dose distribution to the patient's tumor, we simplified the calculation process in keeping with the purpose of the feasibility study (future work with more sophisticated models is described in the Discussion section). We calculate relative dose distributions, normalized to 1, then subsequently scale the result by the planned dose. Portions of the tumor receiving a value of 1 are inside the central "uniform" portion of the radiation beam the whole time. When part of the tumor is in the penumbra of the beam, a value ranging from zero to 1 is assigned to that part (see Fig. 1, top left). To determine what the penumbra should look like, phantom studies were carried out. We varied the shape of the penumbra, calculated the dose, and compared it to the measured dose. The penumbra that produced the best agreement between measured and calculated dose in the phantom was chosen for the dose calculations of the patient.

### *E.1 Phantom studies*

Figure 3 shows a Farmer chamber in a Quasar respiratory motion phantom (Modus Medical Systems, ON, Canada), programmed for sinusoidal motion with a frequency of 13 breaths per minute. The active volume of the Farmer chamber is 20 mm long. The chamber central electrode

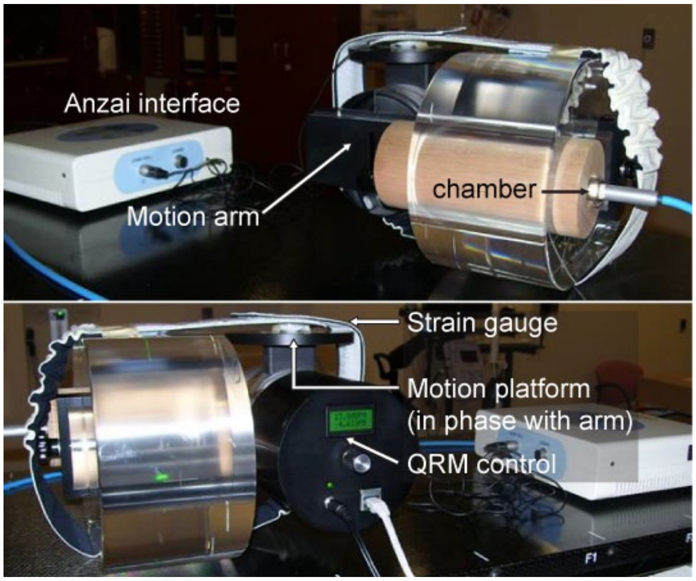

Fig. 3. Quasar respiratory motion phantom.

was coincident with the gantry rotation axis, and the chamber motion was along the gantry rotation axis. A cylinder of cedar wood surrounded the Farmer chamber to simulate a tumor within a lung. The cylinder was attached to the motion arm, which also moved parallel to the gantry rotation axis. A strain gauge was mounted on top of the platform, and a stretchable belt was placed around the phantom and over the strain gauge to provide the variable pressure on the strain gauge. The motion arm was synchronized so that it moves away from the gantry as the platform moves up and the strain gauge pressure increases, thus simulating the inhale phase.

The phantom orientation in the figure seems a little confusing at first, since the belt is actually oriented parallel to the gantry rotation axis. On a patient, the belt would be perpendicular to the gantry rotation axis. In our setup, we could not place the belt in the standard orientation one would expect for a patient. We placed it on the phantom so that it would be stable and would provide the pressure variation reproducibly throughout the experiment. We verified the stability by recording the strain gauge readings for a period of 2 hrs, and we did not observe any drift in the strain gauge "respiratory traces" generated by the phantom motion.

We carefully positioned the phantom in a 5 cm × 5 cm radiation field on an Oncor linear accelerator (Siemens Medical Systems). We adjusted the phantom position along the gantry rotation axis, without any respiratory motion, and measured the dose for 25 MU of a 6 MV beam. We repeated the measurements for several positions in 1 mm increments until we found the maximum dose. There were about 11 positions that had the maximum dose, so we selected the one that was closest to the gantry (the most superior position, or the most cranial position if this were a patient). The resulting setup allowed 1 cm of motion away from the gantry (that is, towards the feet if this were a patient) without an underdose to the chamber.

Once the phantom was positioned, we turned on the motion arm and made several measurements. There were three amplitudes of motion (1 cm, 2 cm, and 3 cm), and ten measurements for each amplitude. We averaged these ten measurements, since slightly different doses can be measured as a result of starting the measurement at a different part of the respiratory phase and one can't predict at what point of the respiratory cycle the radiation beam will turn on. For example, in one measurement, the beam was turned on as soon as the motion arm was fully retracted (simulating full exhale), while in another measurement, the beam was turned on as soon as the motion arm was fully extended (simulating full inhale). The other measurements started at various mid-inhale and mid-exhale phases.

During the phantom measurements, we recorded the strain gauge readings. We did not gate the beam so that we could observe underdoses for the motion amplitudes of 2 and 3 cm and confirm that, for the 1 cm motion amplitude, there was no underdose. We used the strain gauge recordings to calculate the dose that the chamber would receive. We repeated the calculations with various parameters for the length of the uniform portion of the beam and the length and slope of the penumbra region. The calculation method we used for the phantom is the same method we used for the patient (see the next section), except that we only considered one fraction in the calculation. The dose distribution was calculated along the active volume of the chamber; we sliced the active volume into 1 mm disks and calculated the dose for each disk. Finally, since the Farmer chamber only provides one value instead of a dose distribution, we averaged the calculated doses from all of the disks to get a single value for the dose of the chamber.

### *E.2 Patient dose calculation*

To calculate the dose for a given patient's treatment fraction, the strain gauge values recorded during treatment were used to determine the position of the tumor within the beam (Eq. (1)). The tumor was sliced into 1 mm thick disks along the craniocaudal axis. For each disk, at each strain gauge time stamp during beam-on, the disk position was determined from the strain gauge value. The disk was assigned a normalized dose based on the disk position within the beam profile (Fig. 1, bottom of middle and right graphs). In the central region of the profile, it received a value of 1, while in the penumbra region it received a value ranging from 0 to 1, depending on its position. For a given disk, these normalized doses were summed over all time stamps and divided by the number of time stamps. We repeated the process for all disks to produce an average normalized dose distribution for the treatment fraction. These normalized dose distributions were summed over all fractions and multiplied by the planned treatment fraction to determine the total delivered dose distribution in the tumor for the entire course (Fig. 1, bottom).

## F. TCP calculation

Tumor dose distributions were converted to fractional dose-volume histograms (DVHs). To minimize TCP model dependency, TCP values were calculated using the Poisson and Logistic models (Eqs. (2) and (3), respectively):

$$TCP\left(\left\{EQD_2^i\right\}\right) = 0.5^{\sum_{i=1}^{k} v_i \exp\left[\left(2\gamma_{50}/\ln 2\right)\left(1-EQD_2^i/D_{50}\right)\right]} \tag{2}$$

$$TCP\left(\left\{EQD_2^i\right\}\right) = \prod_{i=1}^{k}\left[\frac{1}{1+\left(\dfrac{D_{50}}{EQD_2^i}\right)^{4\gamma_{50}}}\right]^{v_i} \tag{3}$$

where $v_i$ is the fractional volume of the ith subvolume receiving $EQD_2^{i}$ (the equivalent dose in 2 Gy fractions of external beam RT), $\gamma_{50}$ is the normalized dose-response gradient, and the position parameter, $D_{50}$, represents the dose yielding 50% TCP for a given end point. $EQD_2$ was calculated according to Joiner and Bentzen:[(7)]

$$EQD_2^{i} = D_i\left(1+\frac{D_i/n}{\alpha/\beta}\right)\Bigg/\left(1+\frac{2}{\alpha/\beta}\right) \tag{4}$$

where $D_i$ is the cumulative dose from n fractions for the ith subvolume, and the alpha-beta ratio $\alpha/\beta$ = 10 Gy (hence the unit for $EQD_2$ is $Gy_{10}$, with a subscript of 10 to indicate the alpha-beta ratio). Because model parameters depend on treatment technique and end points, TCP reductions were calculated using parameters determined from three-dimensional conformal RT (3D CRT) with local progression-free survival (LPFS) of 12 and 24 months[(8,9)] and image-guided SBRT with LPFS of 36 months.[(10)] The mean doses in $EQD_2$ for 3D CRT were 60 $Gy_{10}$[(9)] and 71 $Gy_{10}$,[(8)] while for SBRT it was 80 $Gy_{10}$.[(10)] Table 2 summarizes these parameters.

Table 2. Tumor control probability (TCP) model parameters.

| *Radiotherapy (RT) Technique* | *Reference* | *Mean Dose, $EQD_2$ ($Gy_{10}$)* | *LPFS (months)* | *$D_{50}$ (Gy)* | $\gamma_{50}$ |
|---|---|---|---|---|---|
| 3D Conformal RT | Willner et al.[(9)] | 60 | 12 | 65.2 | 1.2 |
| | | | 24 | 74.5 | 3.5 |
| | Martel et al.[(8)] | 71 | 12 | 64 | 1.3 |
| | | | 24 | 72 | 2 |
| SBRT | Guckenberger et al.[(10)] | 80 | 36 | 70.5 | 1.4 |

SBRT = stereotactic body radiotherapy; LPFS = local progression-free survival; $EQD_2$ = radiobiological-equivalent dose in 2 Gy fraction of external-beam radiotherapy.

Table 2 is based on patients with various stages of NSCLC (stage I to IV). To account for the heterogeneity of tumor stages (i.e., large interpatient variations), we used the population-based values for the TCP dose-response slope parameter (a lower $\gamma_{50}$ = 1.2–3.5). The dose-response slope increases as interpatient variations decrease.[11] For a single patient, there is no interpatient variation, so we need to use a higher dose-response slope. To estimate the TCP reduction for a given patient, we used a higher $\gamma_{50}$ (= 8) with the available $D_{50}$ values in Table 2. Because $D_{50}$ values are obtained from the same tumor stage group, their combination with a high $\gamma_{50}$ (= 8) simulates a population with minimal interpatient variations. While a high $\gamma_{50}$ (= 8) is somewhat of an artificial construct, it allows us to estimate what could happen to a single patient.

## III. RESULTS

### A. Dose calculation model

In the phantom studies, we experimented with a number of parameters to adjust the length of the uniform central field, the length of the penumbra, and the slope of the penumbra. The results we describe here correspond to the set of parameters that gave us the best agreement between measured and calculated values for the dose received by the farmer chamber. The normalized dose received by the tumor is 1 for positions less than the craniocaudal length of the ITV. Beyond the ITV, the normalized dose decreases to zero at a rate of 0.33 per millimeter. For block margins greater than 5 mm, the normalized dose in the extra margin is also 1. Using the parameters described here, the difference between calculated and measured doses is ≤ 0.3% for motion up to 3 cm. Calculated doses for 1, 2, and 3 cm of motion were 100%, 93.5%, and 79.7% of the planned dose, respectively. The corresponding measured values using the setup in Fig. 3 were 99.7%, 93.6, and 79.6%.

### B. TCP reductions

Table 3 shows the TCP reductions for five NSCLC patients. For the same patients, Fig. 4 shows the amount of motion that exceeded the planned motion (i.e., E = A - P, where *E* is the excess motion, *A* is the total displacement of the tumor from its resting position at exhale, and *P* is the margin that was used in the treatment plan to account for motion). The TCP was reduced by as much as 11.9% for patients with E > 5 mm. The TCP reductions varied with parameter and model choices. Patient 5 showed reductions greater than 6.6% for all parameter and model choices listed in Table 2.

The analysis of the previous paragraph used parameters that are appropriate only when one considers an entire population. For a given individual, a higher $\gamma_{50}$ is possible. If we repeat the analysis of the previous paragraph, but use $\gamma_{50}$ = 8, TCP reductions for patients with E > 5 were as much as 48.6%, using the logistic model. For very low doses, the Poisson model produced unrealistic TCP values, yielding a TCP of zero in some cases (reductions ranged from 0% to 66.7%). A more reasonable result is that the TCP reduction could be up to 48.6% for a given patient, though even that number is only an estimate because our choice for $\gamma_{50}$ = 8 is somewhat artificial (we can only state that it could be higher than the values calculated for the entire population). Patients with E < 5 mm had negligible TCP reductions (≤ 0.3%) using the parameters in Table 2, while for a given patient ($\gamma_{50}$ = 8 and $D_{50}$ values in Table 2) the TCP reduction was ≤ 1.5%. The take-home message for a given individual, regardless of the parameter choices, is that we need to limit excess motion to less than 5 mm.

Because TCP calculations consider the dose delivered for an entire treatment course that could consist of several fractions, it is instructive to determine the effect of repeating the worst case motion over the entire treatment. For Patient 3, the repetition increased the TCP reductions by a factor of 2 to 3, depending on the model parameters (from a range of (1.7, 8.9) to (3.8, 29.7)). Hence, with fewer fractions, it could be very critical to verify that intrafraction motion matches the planned motion. However, with fewer fractions we tend to use higher fraction doses.

Table 3. The expected TCP reductions.

| Parameters | Patient | Poisson TCP Model (%) Planned | Delivered | Reduction | Logistic-TCP Model (%) Planned | Delivered | Reduction |
|---|---|---|---|---|---|---|---|
| | 1, 2[a] | 91.5 | 91.5 | 0 | 90.4 | 90.4 | 0 |
| Willner et al.[9] | 3 | 72.9 | 71.1 | 1.8 | 72.7 | 71 | 1.7 |
| 12 months | 4 | 44.9 | 44.7 | 0.2 | 44.9 | 44.8 | 0.1 |
| | 5 | 50.5 | 43.5 | 7 | 50.5 | 43.7 | 6.8 |
| | 1, 2[a] | 98.7 | 98.7 | 0 | 99.1 | 99.1 | 0 |
| Willner et al.[9] | 3 | 72 | 63.1 | 8.9 | 73.1 | 65.9 | 7.2 |
| 24 months | 4 | 2.9 | 2.8 | 0.1 | 7.9 | 7.7 | 0.2 |
| | 5 | 9.5 | 0.6 | 8.9 | 14.1 | 7.5 | 6.6 |
| | 1, 2[a] | 93.6 | 93.6 | 0 | 92.6 | 92.6 | 0 |
| Martel et al.[8] | 3 | 76.2 | 74.4 | 1.8 | 76.1 | 74.3 | 1.8 |
| 12 months | 4 | 46.9 | 46.7 | 0.2 | 46.9 | 46.7 | 0.2 |
| | 5 | 53 | 45.1 | 7.9 | 53 | 45.4 | 7.6 |
| | 1, 2[a] | 94.8 | 94.8 | 0 | 95 | 95 | 0 |
| Martel et al.[8] | 3 | 69.4 | 65.9 | 3.5 | 69.9 | 66.4 | 3.5 |
| 24 months | 4 | 22.7 | 22.4 | 0.3 | 24.4 | 24.1 | 0.3 |
| | 5 | 31.1 | 19.2 | 11.9 | 31.9 | 23.2 | 8.7 |
| | 1, 2[a] | 90.3 | 90.3 | 0 | 89.8 | 89.8 | 0 |
| Guckenberger et al.[10] | 3 | 66.9 | 64.6 | 2.3 | 67 | 64.7 | 2.3 |
| 36 months | 4 | 33.4 | 33.2 | 0.2 | 33.7 | 33.5 | 0.2 |
| | 5 | 39.7 | 31.7 | 8 | 39.8 | 32.7 | 7.1 |

[a] Patients 1 and 2 have very similar excess motion and fractionation schemes, resulting in essentially identical TCP values. They are shown on one line for convenience.

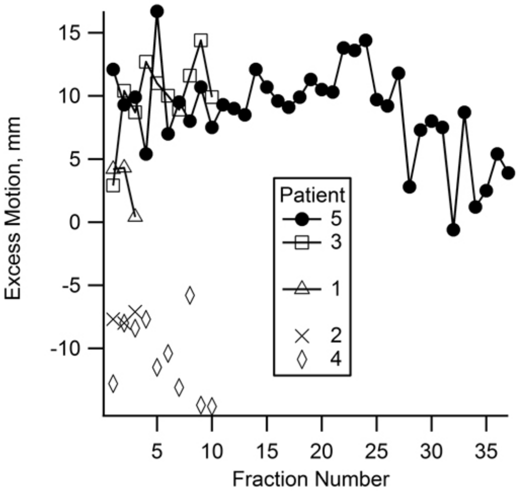

Fig. 4. Variation in treatment motion displacements for five patients. A positive value for excess motion means that the patient's tumor had more motion on the given treatment fraction than the motion at the time the planning 4D CT was acquired. A negative value for excess motion means that the patient's tumor moved less than the motion margin that was used in the plan.

If Patient 3 repeated his worst case motion for three fractions, but received a fraction dose of 16 Gy, the TCP reduction would be reduced, ranging from 2.8% to 10.4%. Note also that the TCP is much higher than that of the 10-fraction plan without excess motion.

On the other hand, a patient with a suboptimal fractionation scheme will have very low TCP values (Patient 4), even if you don't include any dose reduction effects due to motion. When the TCP is so low to begin with, reducing the TCP by considering motion in the dose calculation will not change the clinical outcome.

## IV. DISCUSSION

Our calculations assumed that setup errors used the entire PTV setup margin. CBCT imaging could reduce the setup error below these margins. Unused portions of these margins could supplement motion margins and reduce the impact of excessive motion. However, it is difficult to estimate how much of the setup margin can be used as additional motion margin. For example, Patient 5 does not have an explicit setup margin added to the ITV (i.e., the ITV was called the PTV). This was a matter of convenience because the patient had two treatment regions: the mediastinum and the lung nodule. In the process of contouring and combining these regions, the margin created for the lung nodule, in effect, is used to account for setup errors and excess motion. A more realistic dose calculation would require knowing the tumor location after the patient was shifted to correct setup errors. The calculation could also be improved by using the exact shape and motion of the tumor.

To improve our dose calculations, one could use the motion information derived from the analysis of the strain gauge and CBCT data described here with a few modifications. We have found that using a quadratic rather than a linear fit between the strain gauge and the respiratory traces is more realistic, and that the curve fit parameters for the inhale portion of the respiratory trace is different from those for the exhale portion. By using these phase-dependent quadratic fits, we can determine what proportions of the delivered dose occurred at various tumor positions, and the dose could be calculated using the nearest phase of the 4D CT dataset obtained for treatment planning. For tumor positions beyond the extreme position in the full inhale phase of the 4D CT dataset, one could use the full inhale phase and move the GTV to the required position. The dose distributions in the tumor for the entire treatment course would then be a weighted sum of the dose distributions determined at each tumor position. The weights would be derived from the strain gauge and CBCT analysis of tumor positions recorded over all treatment fractions. Calculating the dose distributions in a commercial treatment planning system also takes care of another assumption in the simple calculation model — that the tumor does not change shape (e.g., get compressed or stretched) or size as it moves. By using the 10 phases of the 4D CT dataset, one can define the tumor shape and size for each phase. The motion can be tracked based on the centroid of the tumor, since that will average out any uncertainties involved in relating the strain gauge values to tumor position.

The proposed 4D dose calculation is similar to the approach described by Vinogradskiy et al.,[12] with the exception that their calculation did not account for actual tumor motion at the time of treatment. Other authors have also done only prospective 4D calculations based on the planning data, using probability density functions,[13] mean patient density representations,[14] or stochastic models to describe respiratory motion.[15] To date, no retrospective analysis of actual delivered dose distributions in gated treatments has been done. Our study represents a potentially better approximation of the delivered dose, and future studies could combine our methods for recording tumor motion with 4D dose calculation techniques.

Other assumptions in our method could also be improved. For example, the diaphragm-to-tumor motion ratio assumes that the relationship of the tumor to the diaphragm is constant over all fractions. While the constancy of the relationship might be true over a few fractions, it is possible that, as the tumor responds to treatment, the mechanical properties in the neighborhood of the tumor may change and the diaphragm-to-tumor motion ratio could change. One could sort the projections of the CBCT into a full-exhale subset and a full-inhale subset and reconstruct the subsets to get a 3D CBCT at full exhale and at full inhale. The diaphragm-to-tumor motion ratio could then be verified on a daily basis, and the calculations could use the ratio specific for each fraction. The calibration of the strain gauge to the diaphragm motion is already done for each fraction, so the method accounts for the daily changes in their relationship. We have done preliminary studies[3,16] to show the feasibility of tracking the diaphragm from CBCT projections, reconstructing full exhale and inhale 3D CBCTs, relating them to strain gauge values, and determining tumor motion. During these studies, we also confirmed the approximate validity of our method described in support of Eq. (1), and the potential improvements from using the results of our diaphragm tracking studies.

Even if all these sources of uncertainty were resolved, a fundamental assumption in Eq. (4) will still be violated. It is assumed that the dose received by a subvolume is the same from one fraction to the next, but Fig. 4 shows that the amount of excess motion varies and, hence, the underdose in a given subvolume is not constant across all treatment fractions. Equation (4) is only an approximation. However, model parameters in the literature are based on outcomes resulting from treatments that include the effects of subvolume dose variations. Hence, using model parameters for 3D CRT treatments in our study could help to account for the subvolume dose variation across treatment fractions.

While Guckenberger et al.[10] has TCP model parameters for patients treated in the image guided RT era with hypofractionation, no TCP model parameters exist that take into account the actual dose distributions delivered to the patient. In order to develop such models, we need to determine the motion that occurred while the patient was being treated and account for that motion when calculating dose distributions in the tumor. Our work shows that it is feasible to estimate delivered doses by monitoring the motion throughout the entire treatment. Although we had only five patients on the study, our goal was to show that there are individuals for whom the consideration of excess motion during gated treatment is important. While the dose calculation methodology in our study was simple, it provided a proof of concept. Future research would involve taking the observed treatment motion and combining dose distributions calculated for various tumor positions in a commercial treatment planning system. The contributions from the dose distributions for each tumor position could be weighted according to how many MUs were delivered at each position. The resulting dose distributions could then be combined with the corresponding local progression free survival data to produce better TCP models.

Mechalakos et al.[5] showed that it is important to consider motion even for nongated 3D CRT. Using pretreatment fluoroscopic sequences from seven patients, they developed a breathing motion model. They randomly chose a breathing trace from the model to shift dose distributions within the tumor, according to respiratory induced displacements. To improve the statistics, they performed the random selections 500 times for each patient, resulting in 500 dose distributions with varying amounts of motion. In some cases, they found slight increases in the TCP since they allowed setup variations to counteract breathing motion. For the cases where they reported decreases, these were on the order of 1% to 5%. For their worst case motion, the TCP reduction was in the range of 8% to 17%. While these values appear to be consistent with our observations, the exact nature of their TCP model and fractionation scheme was not specified, so a direct comparison is not possible. Their simulations were based on randomly sampling motion from the model developed with seven patients. Their method does not account for systematic factors that any one given patient might experience to change their tumor motion during the course of treatment.

In our study, Patient 5 had 37 fractions, and his tumor motion pattern changed from one day to the next, with a trend suggesting the excess motion was decreasing over time. However, his respiratory motion during treatment was greater than the motion included in the initial treatment plan for 36 of the 37 fractions. Systematic factors, such as increased anxiety at the time of 4D CT imaging, changes in tumor size, changes in medication, and/or other changes in physiological state, could explain the changes in tumor motion. The presence of systematic factors reminds us that a TCP analysis must include the tumor motion recorded at the time of treatment.

Other authors have also noted the importance of managing respiratory motion for every fraction. Mechalakos et al.[5] reported the dosimetric and radiobiological impact of breathing motion in terms of tumor coverage for NSCLC patients. The dose to 95% of the GTV ($D_{95}$) changed an average of -9.8% ± 10.1% and the TCP changed by -8.1% ± 9.1% for heavy breathers. They reported that respiratory gating can decrease the chances of reducing $D_{95}$ by 10% or more. Without gating, the $D_{95}$ was reduced by at least 10% for about 40% of the heavy breathers. With gating, only 10% of the heavy breathers experienced the $D_{95}$ reduction of 10%

or more. However, even for gated patients, the choice of gating methods can affect the tumor doses. Riboldi et al.[17] compared tumor tracking uncertainties of amplitude- and phase-based respiratory gating using a motion probability density function analysis. They reported that amplitude-based gating resulted in reduced errors when compared to phase-based gating. However, they only analyzed the results for two patients and their report did not include active or passive breath-hold techniques. Regardless of the gating technique, the likelihood of a geometric miss could be reduced if the tumor motion can be assessed on a daily basis, or if the tumor motion can be reliably reproduced. Kubo et al.[18] used fluoroscopic studies in conjunction with the real-time position management respiratory gating system (RPM) to synchronize target motion with the external breathing motion waveform, while Goossens et al.[19] reported improved tumor motion reproducibility as a result of using audio-visual coaching techniques.

Intuitively, one might expect that improving reproducibility would be even more important for few fraction treatments, where you have fewer chances to make corrections if an error is found. However, it turns out that Patient 3 would have benefitted from hypofractionation, even in the presence of worst case motion. It could be that high doses may be compensating for our inaccuracies in target localization. Despite that, it is still important to improve our localization accuracy, since the improvement could make it possible to conduct dose de-escalated hypofractionation studies. Other authors have performed computer simulations to show the feasibility of adapting the gating window based on MV imaging; they were able to reduce the residual motion to 3 mm.[20]

## V. CONCLUSIONS

It is feasible to calculate delivered doses and their corresponding TCP reductions for gated radiotherapy. The phantom studies showed excellent agreement between calculated and measured doses, with less than 0.3% error for motion up to 3 cm. Motion in excess of planned motion for gated radiotherapy can cause up to 11.9% reductions in TCP when the excess motion is 5 mm or more. When using the nonpopulation-based gradient value ($\gamma_{50}$ = 8), TCP reductions of up to 48.6% were observed. When worst case motion is repeated, TCP reductions can increase by a factor of 2 to 3, while excess motion on the order of 3 mm or less has a negligible effect on the TCP. In the absence of adaptive gating strategies or daily 4D verification of tumor motion and the calibration of tumor motion monitors, larger margins may be justifiable. However, by applying daily tumor motion verification and adjusting the gating window, even hypofractionated regimens could benefit, potentially opening the doors for dose de-escalation and reducing healthy tissue dose.

## Supporting information

Supplementary Material
