# Supplementary material for: Tumor control probability reduction in gated radiotherapy of non‐small cell lung cancers: a feasibility study
Source: J Appl Clin Med Phys. 2014 Oct 16;16(1):8–21. doi: 10.1120/jacmp.v16i1.4444 (PMC5689977; doi:10.1120/jacmp.v16i1.4444)
Supplement: Supplementary file 1 — Supplementary Material [file ACM2-16-008-s001.docx]

3. Chen M, Siochi RA. Diaphragm motion quantification in megavoltage cone-beam CT projection images. Med Phys 2010;37:2312-2320.

16. Chen M, Siochi RA. Feasibility of Using Respiratory Sorted Mega Voltage Cone Beam Computed Tomography to Measure Tumor Motion. J Appl Clin Med Phys 2011; 12: 201-212.
